# The Role of Ethylene in Plants Under Salinity Stress

**DOI:** 10.3389/fpls.2015.01059

**Published:** 2015-11-27

**Authors:** Jian-Jun Tao, Hao-Wei Chen, Biao Ma, Wan-Ke Zhang, Shou-Yi Chen, Jin-Song Zhang

**Affiliations:** State Key Lab of Plant Genomics, Institute of Genetics and Developmental Biology, Chinese Academy of Sciences, Beijing, China

**Keywords:** ethylene, salinity stress, MHZ, NtTCTP, negative feedback

## Abstract

Although the roles of ethylene in plant response to salinity and other stresses have been extensively studied, there are still some obscure points left to be clarified. Generally, in *Arabidopsis* and many other terrestrial plants, ethylene signaling is indispensable for plant rapid response and tolerance to salinity stress. However, a few studies showed that functional knock-out of some ACSs increased plant salinity-tolerance, while overexpression of them caused more sensitivity. This seems to be contradictory to the known opinion that ethylene plays positive roles in salinity response. Differently, ethylene in rice may play negative roles in regulating seedling tolerance to salinity. The main positive ethylene signaling components MHZ7/OsEIN2, MHZ6/OsEIL1, and OsEIL2 all negatively regulate the salinity-tolerance of rice seedlings. Recently, several different research groups all proposed a negative feedback mechanism of coordinating plant growth and ethylene response, in which several ethylene-inducible proteins (including NtTCTP, NEIP2 in tobacco, AtSAUR76/77/78, and AtARGOS) act as inhibitors of ethylene response but activators of plant growth. Therefore, in addition to a summary of the general roles of ethylene biosynthesis and signaling in salinity response, this review mainly focused on discussing (i) the discrepancies between ethylene biosynthesis and signaling in salinity response, (ii) the divergence between rice and *Arabidopsis* in regulation of salinity response by ethylene, and (iii) the possible negative feedback mechanism of coordinating plant growth and salinity response by ethylene.

## Introduction

Salinity has extensive negative effects on plant growth, including ion toxicity, osmotic stress, oxidative stress, and nutrient deficiency (Chinnusamy et al., 2006; Zhu, 2007). These hazards lead to growth inhibitory, crop yield reduction, and even death under prolonged high salinity condition. For improper irrigation and drainage, back flow of seawater, abuse of chemical fertilizer, and some other reasons, more and more arable land tends to be threatened by salinity. Salinity will usually lead to soil sodicity and alkalinity. Therefore, salinity stress has turned out to be one of the major factors limiting the sustainable development of agriculture. For a long time, people have focused on elucidating the physiological effects of salinity stress on plants, dissecting genetic components responsible for salinity stress response and resistance, and improving plant tolerance to salinity mainly through hybridization and gene transformation. Nowadays, studies at integrative levels become prevalent to uncover the intrinsic mechanism for plant rapid responses and self-modulation of vegetative and reproductive growth to adapt to salinity condition.

Throughout the life cycles of plants, phytohormones play vital roles in controlling the interaction between plants and environments, including plant responses to salinity stress. One of the phytohormones involved in salinity response is ethylene, which is also regarded as a stress-hormone besides its roles in regulation of plant growth and development (Abeles et al., 1992). Levels of ethylene and its direct precursor ACC could be obviously induced by salinity and other abiotic stresses in many plant species (Morgan and Drew, 1997). Compared to the glycophyte *Arabidopsis thaliana*, two halophytes *Cakile maritima* and *Thellungiella salsuginea* accumulated more ACC in both leaves and roots under high salinity (Ellouzi et al., 2014). In soybean, a research using 2-DE gel analysis found that several components of ethylene biosynthesis in the salt-tolerant genotype Lee 68 were more abundant than that in the salt-sensitive genotype Jackson under salinity stress (Ma et al., 2012). Application of ethylene or ACC could improve plant tolerance to high salinity (Cao et al., 2007), largely through enhancing the expression of reactive oxygen species (ROS) scavengers (Peng et al., 2014a). Further studies based on gene mutation and transformation analysis elucidated that the whole ethylene biosynthesis and signal transduction pathway are involved in plant response and adaptation to salinity. Generally, promotion of ethylene biosynthesis and signal transduction could enhance plant tolerance to salinity, while inhibition of it leads to increased plant sensitivity to salinity.

However, some other works showed that ACC may play a negative role in regulation of tomato seedling growth under salinity (Albacete et al., 2009). In tomato, ACC in leaves was increased prior to Na^+^ accumulation, and was coincident with the onset of oxidative stress and leaf senescence under salinity stress (Ghanem et al., 2008). Although these results did not show the direct effects of ACC on plant response to salinity, and some other works revealed that ethylene is not the primary factor in salinity-induced plant growth inhibition (Shibli et al., 2007), it is still possible that ethylene may play a subtle negative role in plant response to salinity, at least at a certain growth stage. A further discussion is therefore necessary for us to understand the actual roles of ethylene in plant response to salinity.

In this review, we summarized and discussed roles of the whole ethylene biosynthesis and signaling pathway in plant response to salinity stress. The interactions between ethylene and ABA, jasmonic acid (JA) and some other signaling molecules, and the cross-talks between plant response to salinity and response to nutrient deficiencies were also compared. Some possible divergent roles of ethylene in different plant species were discussed as well.

## Ethylene Biosynthesis and Salinity Stress

Ethylene is a simple gaseous hormone which plays multiple roles in regulation of plant growth and development, and also serves as a key modulator between plant response to environmental stresses and normal growth (Abeles et al., 1992). Under salinity and some other stresses, ethylene production is quickly stimulated (Morgan and Drew, 1997). In fact, several key steps of ethylene biosynthesis could be affected by salinity and other stresses. Ethylene in plant is synthesized through three enzymatic reaction steps: methionine is converted to *S*-adenosyl-methionine (*S*-AdoMet) by *S*-AdoMet synthetase; then the direct precursor of ethylene ACC is synthesized from *S*-AdoMet by ACS (ACC synthase); and finally ethylene is produced through the oxidation of ACC by ACO (ACC oxidase) (reviewed in Lin et al., 2009). *S*-AdoMet is also the precursor for the synthesis of polyamines, which also plays a role in plant response to biotic and abiotic stresses. The by-product MTA (5′-methylthioadenosine) from the second step is recycled to methionine through the Yang Cycle to maintain a stable methionine pool even when ethylene is rapidly synthesized (Yang and Hoffman, 1984; Kende, 1993).

As a rate-limiting enzyme, ACS is the major target for regulation of ethylene production under stresses (Yang and Hoffman, 1984; Kende, 1993; Bleecker and Kende, 2000; Wang et al., 2002). There are eight functional *ACS* genes in *Arabidopsis*. They have redundant role in ACC biosynthesis (Tsuchisaka and Theologis, 2004; Tsuchisaka et al., 2009), while each with unique function in the regulation of plant growth and development. The regulation of *ACSs* under stress occurs at both transcriptional and post transcriptional levels. Under salinity, transcripts of *ACS2* and *ACS7* in *Arabidopsis* were increased dramatically (Achard et al., 2006). Through GUS staining, *Arabidopsis ACS5* and *ACS7* promoters were found to be induced by salinity (Wang et al., 2005). A more systematic research revealed that the MAPK cascade-induced by stress might activate WRKY33 which then promoted the expression of *ACS2/ACS6* genes in *Arabidopsis* (Li et al., 2012). In tobacco, the transcripts of *ACS1* were induced by salinity (Cao et al., 2006). In cotton, a series of *ACSs* were up-regulated under both short- and long-time salinity condition (Peng et al., 2014b). Recent work in *Arabidopsis* found that four *ACSs* (*ACS2*, *ACS6*, *ACS7*, and *ACS8*) were induced by high salinity, while a moderate low salinity pretreatment (known as salt acclimation) alleviated this induction (Shen et al., 2014). This result provides us a cue that promotion of ethylene production by a strong and sudden salinity stress might have multiple effects for plant response to salinity. It seems that no extra ethylene was needed for the process of salt acclimation, because there were no changes on the expression of genes related to ethylene biosynthesis (Shen et al., 2014). Four *ACSs* were up-regulated no matter under NASS (non-acclimated salt stress) or SASS (salt acclimated salt stress; Shen et al., 2014), indicating that promotion of ethylene production is still necessary for plant adaption to the stress condition. Nevertheless, transcripts of ACSs under SASS were less than those under NASS (Shen et al., 2014). These results suggest that a tight control of ethylene biosynthesis might be important for plant response and adaption to salinity stress.

Besides transcriptional regulation, some *ACSs* are also regulated post-transcriptionally under salinity, mainly through stress-activated MAPK (mitogen-activated protein kinase) cascades which phosphorylate ACSs protein and then prevent them from 26S proteasome-mediated degradation. Under biotic and abiotic stresses, *Arabidopsis* MPK6 was activated rapidly to phosphorylate ACS2 and ACS6 to elevate ethylene production (Liu and Zhang, 2004). In tomato, it was shown that calcium-dependent protein kinases (CDPKs)-mediated phosphorylation stabilized ACS2, leading to increased ACC content in stressed tissues (Tatsuki and Mori, 2001; Kamiyoshihara et al., 2010). In *Arabidopsis*, loss-of-function of MPK6 diminished the effects of salt acclimation, leading to more sensitivity to high salinity (Shen et al., 2014).

Another key ethylene biosynthesis enzyme ACO is also regulated by salinity. Under high salinity, both ACC content and ACO activity were increased to enhance ethylene release in *Cicer arietinum* root (Kukreja et al., 2005). In cotton, several *ACOs* were up-regulated under both short- and long-time salt treatment (Peng et al., 2014b). From these results, we can propose that plants under salinity and other abiotic and biotic stresses tend to produce more ethylene mainly through enhancing ACSs and ACOs. Nevertheless, transcripts of *ACO1* in wheat were decreased under salinity and other abiotic stresses (Chen et al., 2014).

Although ACSs and ACOs are usually up-regulated under salinity, these key enzymes for ethylene biosynthesis may play negative roles in plant salinity-response. In *Arabidopsis*, loss-of-function of ACS7 conferred less ethylene emission, promoted vegetative growth and enhanced tolerance to salinity (Dong et al., 2011). Constitutive expression of wheat *ACO1* in *Arabidopsis* led to salinity-sensitivity, possibly through increasing the expression of *AtMYB15* while suppressing some stress-responsive genes like *AtRAB18*, *AtCBF1*, and *AtCBF3* (Chen et al., 2014). Expression of MKK9 in transgenic plants activated the endogenous MPK3 and MPK6 kinases, promoted the synthesis of ethylene and camalexin, and finally conferred increased sensitivity to salinity, whereas loss-of-function mutant *mkk9* showed enhanced tolerance to salinity (Xu et al., 2008). Recent work in rice found that a lectin receptor-like kinase SIT1 (salt intolerance 1) mediated salinity sensitivity through regulation of ethylene homeostasis (Li et al., 2014a). In presence of salinity stress, SIT1 was activated rapidly to phosphorylate MPK3/6 and then promote ethylene production (Li et al., 2014a). Overexpression of SIT1 in rice and *Arabidopsis* both conferred increased sensitivity to salinity, while loss-of-function of MPK3/6 alleviated this effect (Li et al., 2014a). Expression of SIT1 in rice and *Arabidopsis* also enhanced the ROS accumulation in an MPK3/6- and ethylene signaling-dependent manner (Li et al., 2014a). All indicate a SIT1-MPK3/6 cascade mediates salt sensitivity by regulating ethylene homeostasis. In addition, plant growth-promoting rhizobacteria (PGPR) produced AcdS (ACC deaminase), which facilitated the growth and stress tolerance of hosts via a reduction in levels of ethylene (Ali et al., 2014; Barnawal et al., 2014; Kim et al., 2014). Transgenic *Arabidopsis* expressing AcdS showed reduced sensitivity to exogenous ACC but increased tolerance to high salinity. In contrast, AcdS-silenced *Trichoderma* mutants were less effective in promoting plant tolerance to salinity, indicating that *Trichoderma* can also ameliorated plant growth under salinity stress by decreasing the ethylene biosynthesis in hosts (Brotman et al., 2013).

In contrast to the studies above, some other works showed that ethylene biosynthesis has positive effects on salinity-response. As stated above, MPK6, a key regulator of ethylene biosynthesis under stresses, was necessary for salt acclimation (Shen et al., 2014). That means plants may need MPK6 to stabilize ACSs and maintain a relative high ethylene level to accomplish the salt acclimation. The BTB ubiquitin ligases ETO1, EOL1, and EOL2 could interact with ACS5, promoted its ubiquitination and then accelerated its degradation (Christians et al., 2009). The expression of *ETOL1* in rice was induced under 200 mM NaCl treatment (Du et al., 2014). In *Arabidopsis*, loss-of-function of ETO1 increased ethylene production and improved seedling tolerance to soil-salinity. Lack of ETO1 reduced root Na^+^ influx and so restricted root-to-shoot delivery of Na^+^, and these effects were associated with increased RBOHF-dependent ROS accumulation in root stele. In addition, loss-of-function of ETO1 enhanced the tissue K^+^ status through an RBOHF-independent manner associated with increased transcripts of the K^+^-transporter *HAK5* (Jiang et al., 2013).

From these results, a definitive conclusion could not be drawn as to whether ethylene biosynthesis plays positive or negative roles in plant response to salinity. The discrepancy in the role of ethylene biosynthesis in salt response may be due to that, usually a single member of genes in the ethylene biosynthesis pathway, but not the whole status, was evaluated. And the optimal ethylene level for normal plant growth may be varied at different stages and in different plant species. From an evolutionary perspective, the induction of ethylene under stress condition should have some advantages for plant survival. In fact, pretreatment with ACC enhanced the salinity-tolerance of *Arabidopsis* seedlings (Peng et al., 2014a). We propose that ethylene indeed plays a positive role in the early stage of plant self-adjustment or salt acclimation for better survival under high salinity stress. After the stage of self-adjustment, excessive ethylene in plants will inhibit plant growth and development, which is disadvantageous for plants to survive under high salinity stress. Additionally, various ethylene receptors, whose functions are negatively regulated by ethylene, may also “neutralize” the ethylene role in salt stress. A homeostasis between ethylene and its receptors may facilitate plant survival under salinity stress.

There are multiple ACSs and ACOs members with functional redundancy on ethylene synthesis. Single mutation of one member only decreased ethylene emission in a limited level, and this may have little effect on salt acclimation. However, under high salinity, single mutation of ACS or ACO might decrease the induction of ethylene and alleviate excessive ethylene-induced growth inhibition, and hence keep higher growth potential for plants. Another possible reason is the competition for *S*-AdoMet as the precursor between ethylene and PA (polyamines) biosynthesis. Decreasing the ACS and ACO activity in tobacco could promote the biosynthesis of PA, and so enhance plant tolerance to abiotic stresses including high salinity through PA-mediated pathway (Wi and Park, 2002). Additionally, ACC itself might act as a signaling molecule in plants (reviewed in Yoon and Kieber, 2013), and so possibly mediates some unknown salinity response pathways. Further research based on analysis of multiple mutants of ACSs or treatments with different ethylene biosynthesis inhibitors will shine a light on the mechanisms involved.

## Ethylene Signaling and Salinity Stress

Based on the classical “triple response” (inhibited hypocotyl and root elongation, enhanced horizontal growth and exaggerated apical hook of etiolated seedlings), a series of ethylene response mutants were characterized in *Arabidopsis*. The ethylene signal transduction model was then established through genetic and biochemical analysis of these mutants. Main components of this model include five ethylene receptors, a negative regulator CTR1, a key positive regulator EIN2, primary transcription factors EIN3/EILs and many downstream ethylene-response factors. In normal condition, ethylene receptors interact with and activate CTR1, which then phosphorylates EIN2 and prevents its translocation into nucleus, therefore inhibits ethylene signal transduction. When developmental or environmental signals induce ethylene production, ethylene binds with receptors to inhibit their interaction with CTR1, and CTR1 is inactivated, leading to the dephosphorylation and cleavage of EIN2. Then the C-terminus of truncated EIN2 is translocated into nucleus to stabilize downstream transcription factors EIN3/EILs (Ju et al., 2012; Qiao et al., 2012; Wen et al., 2012). Finally, transcriptions of downstream ethylene response factors are activated by EIN3/EIL1 and lead to extensive ethylene responses. In this pathway, EIN2 is regulated by ETP1/ETP2 mediated protein turnover (Qiao et al., 2009) and EIN3 is regulated by EBF1/EBF2-dependent ubiquitination and degradation (Guo and Ecker, 2003; Potuschak et al., 2003; Gagne et al., 2004; Binder et al., 2007; An et al., 2010). Ethylene receptors are also regulated by proteasome-mediated protein degradation (Chen et al., 2007; Kevany et al., 2007; Shakeel et al., 2015; Tao et al., 2015). More recently, two groups reported new advances of ethylene signaling. Li et al. (2015a) found that ethylene induced EIN2 to associate with the 3′-UTR of *EBF1/2* mRNA and target *EBF1/2* mRNA to the cytoplasmic processing-body (P-body) through interaction with multiple P-body factors, leading to the stabilization of EIN3/EIL1 and activation of downstream events. Merchante et al. (2015) adopted genome-wide ribosomal footprinting and RNA-seq methods to identify translationally-regulated genes by ethylene, and they found that EIN2 and non-sense-mediated decay proteins UPFs are required for the translational regulation of *EBF1/2*.

As a stress hormone, the whole signaling pathway of ethylene is involved in plant response to salinity stress and possibly mediated the stress signal transduction. First, the expressions of many ethylene signaling genes are regulated by salinity and other stresses. In *Arabidopsis*, the expression of ETR1 was suppressed by osmotic stress including salinity (Zhao and Schaller, 2004). In tobacco, the mRNA quantity of ethylene receptor NTHK1 was dramatically increased under salinity stress (Zhang et al., 2001; Cao et al., 2006, 2007; Zhou et al., 2006). Further research discovered that NTHK1 was regulated by proteasome-mediated degradation, while its interaction with NtTCTP could stabilize itself (Tao et al., 2015). In cotton, several ethylene receptor genes (*ETR1*, *ETR2*, and *EIN4*), ethylene signaling genes (*CTR1*, *EIN3*, *ERF1*, and *ERF2*) and MAPK cascade genes (*MEKK1*-*MKK2*-*MPK4/6*) were all up-regulated under both short- and long-time salt treatments (Peng et al., 2014b). Besides, salt treatment promoted the degradation of EBF1/EBF2 and so enhanced the EIN3 protein accumulation in an EIN2-independent manner (Peng et al., 2014a). Additionally, salinity also promoted the transcriptional activity of EIN3 in an EIN2-dependent manner (Peng et al., 2014a).

Second, based on mutation and transgenic analysis, almost all ethylene signaling components were found to participate in plant response to salinity and other stresses. In *Arabidopsis*, *etr1* loss-of-function mutants showed enhanced tolerance to high salinity, whereas gain-of-function mutant *etr1-1* displayed increased sensitivity to salt stress (Zhou et al., 2006; Cao et al., 2007; Peng et al., 2014a). Overexpression of *NTHK1* in tobacco and *Arabidopsis* led to reduced sensitivity to ethylene but enhanced sensitivity to salinity (Cao et al., 2006). Addition of ACC to the treatment alleviated the salinity sensitivity caused by the overexpression of NTHK1 in *Arabidopsis*, but has no effects on the gain-of-function mutants of ethylene receptors (*etr1-1* and *ein4-1*, Cao et al., 2007). Further truncation and mutation analysis of NTHK1 showed that the transmembrane domain, the kinase domain and the kinase activity were indispensable for its roles in conferring reduced sensitivity to ethylene but enhanced sensitivity to salinity (Zhou et al., 2006; Chen et al., 2009). Besides redundant roles in ethylene perception, each ethylene receptor has some specific roles (reviewed in Shakeel et al., 2013). The specificity of ethylene receptors was reflected partly in their roles in salinity response. In *Arabidopsis*, ETR1 and ETR2 had contrasting roles in seed germination during salt stress, which seems to be mediated by affecting the ABA signaling but independent of ethylene signaling (Wilson et al., 2014). Tobacco subfamily II receptor NTHK1 played stronger roles than the subfamily I receptor ETR1 in regulation of seedling growth and salinity response (Chen et al., 2009). By yeast two-hybrid screening, an ankyrin domain-containing protein NEIP2 was identified to interact with NTHK1 and mediated plant response to salinity stress (Cao et al., 2015).

Besides the receptors, downstream ethylene signaling components also participated in salinity response. Loss-of-function of the key negative ethylene signaling factor CTR1 led to more tolerance to salinity stress (Achard et al., 2006; Peng et al., 2014a), possibly through modulation of shoot Na^+^ and K^+^ homeostasis which was dependent on ETR1-CTR1-regulated ethylene signaling (Jiang et al., 2013). The key positive ethylene signaling factor downstream of CTR1 is EIN2, which is found to confer salinity tolerance. *Arabidopsis* seedlings with loss-of-function of EIN2 became more sensitive to salinity, while overexpression of the C-terminus of EIN2 in *ein2-5* suppressed the salinity sensitivity (Cao et al., 2007; Lei et al., 2011a; Peng et al., 2014a). By yeast two-hybrid screening, a MA3 domain-containing protein ECIP1 was identified to interact with EIN2. Loss-of-function of ECIP1 led to minor enhanced ethylene response and salt tolerance during seedling growth but conferred salt sensitivity during seed germination process (Lei et al., 2011a). EIN3/EILs are the primary transcription factors for ethylene signal transduction from EIN2 to nuclear transcriptional regulation. In *Arabidopsis*, loss-of-function mutant *ein3-1* and the double mutant *ein3eil1* exhibited severe sensitivity to salinity, whereas overexpression of EIN3 enhanced seedling tolerance to salinity (Achard et al., 2006; Lei et al., 2011a; Peng et al., 2014a). Single mutant *ebf1-1* and double mutant *ebf1-1ebf2-1* also showed enhanced tolerance to salinity in an EIN3-dependent manner (Achard et al., 2006; Peng et al., 2014a).

Generally, in plant response to salinity, positive components of ethylene signaling are up-regulated by salinity and play positive roles, whereas negative factors are correspondingly down-regulated and play negative roles. In one word, ethylene signaling is necessary for plant response and adaption to salinity stress. However, ethylene response and signaling in rice, a semi-aquatic plant, seems to be somewhat different from *Arabidopsis* (Yang et al., 2015a). Based on the ethylene “double response” (promotes coleoptile growth but inhibits root elongation of dark-grown etiolated seedlings) in rice, a series of ethylene-response mutants *mhz* (*maohuzi*) were identified (Ma et al., 2010, 2013). These mutants were insensitive to ethylene on root elongation but showed differential responses on coleoptile growth. Unexpectedly, but interestingly, except for MHZ7 (a homolog of EIN2) and MHZ6 (a homolog of EIN3), other MHZs were either novel components with no homologies to ethylene signaling pathway proteins in *Arabidopsis* or cross-talk points interacting with other hormones (Ma et al., 2013), indicating a special feature for ethylene signaling pathway in rice. Both MHZ4 and MHZ5 were involved in ABA biosynthesis, and mediated ethylene-controlled root growth (Ma et al., 2014; Yin et al., 2015). MHZ6/OsEIL1 and OsEIL2 respectively regulated ethylene-controlled root and coleoptile growth of etiolated seedlings. Unlike the positive roles of EIN2 and EIN3 in salinity response in *Arabidopsis*, MHZ7/OsEIN2, MHZ6/OSEIL1, and OsEIL2 exhibited the opposite effects on the salinity tolerance of rice seedlings. Functional knock-out of MHZ7/OsEIN2, MHZ6/OSEIL1, or OsEIL2 led to enhanced salt tolerance, while overexpressing each of them increased seedling sensitivity to salinity (Yang et al., 2015b). Na^+^ measurement and downstream gene analysis revealed that MHZ6/OSEIL1 and OsEIL2-regulated salinity responses were mediated through controlling the expression of *OsHKT2;1*, a Na^+^ transporter gene, and the homeostasis of Na^+^ in plants (Yang et al., 2015b). EMSA and luciferase assay showed that both MHZ6/OSEIL1 and OsEIL2 could bind to the promoter region of *OsHKT2;1* and promoted its transcription (Yang et al., 2015b). Particularly, there is no homolog of OsHKT2;1 in *Arabidopsis*, indicating that OsHKT2;1 may be the major reason of difference between rice and *Arabidopsis* on the role of ethylene in salinity response. It is likely that rice adopts the ethylene signaling pathway to activate *OsHKT2;1* expression and Na^+^ uptake for cell ion homeostasis in water-saturated soil. However, this mechanism would lead to the excessive Na^+^ uptake under high salinity condition and result in the salt sensitivity. Further studies on the roles of other rice ethylene signaling components in salinity response would generate more valuable data for elucidating the differences between terrestrial plants and aquatic/semi-aquatic plants on salinity response.

## Downstream Events in Ethylene Mediated Salinity Response

The main ethylene signaling components downstream of EIN3 are ERFs (ethylene-responsive element binding factors), which are plant-specific transcription factors responsible for nuclear transcriptional regulation of a series of effectors related to ethylene response. In *Arabidopsis*, the three classes of ERFs, with either transcriptional-activation or -repression activities, were differentially regulated by ethylene and abiotic stresses (Fujimoto et al., 2000). EIN3/EILs could bind directly to the promoter of *ERF1* and activate its expression. Similar to *EIN3*-overexpression in transgenic plants, expression of *ERF1* also activated a variety of ethylene response genes and led to constitutive ethylene response (Solano et al., 1998). The expressions of *AtERF1*, *AtERF2* and *AtERF5* were all induced by ethylene treatment in an EIN2-dependent manner. The induction of *AtERF3* and *AtERF4* by high salinity stress was regulated by EIN2-mediated ethylene signaling (Fujimoto et al., 2000). Further analysis showed that *ERF1* was highly induced by salinity and drought stress in an ethylene and JA signaling-dependent manner. Overexpression of *ERF1* enhanced plant tolerance to salt, drought and heat stress (Cheng et al., 2013). Although overexpression of many *ERFs* genes could enhance salt tolerance, most of them seem to be independent of ethylene signaling. Based on microarray analysis, three *ERFs* genes in *Arabidopsis*, named as *ESE1* to *ESE3*, were found to be ethylene- and salt-inducible. Among them, *ESE1* was positively regulated by ethylene signaling at transcriptional level, and was downstream of EIN3/EIL1. Further analysis revealed that EIN3 could physically bind to the promoter of *ESE1* and activate its transcription. Then, ESE1 bound to the promoters of *RD29A*, *COR15A* and some other salinity-responsive genes to promote their transcription, and eventually enhanced plant tolerance to salinity (Zhang et al., 2011b).

Besides ERFs, some other factors were also found to be responsive to ethylene signaling and involved in salinity response. A NAC-type transcription factor gene *AtNAC2* was induced by ACC, ABA, NAA, and salinity treatments. The salt induction of *AtNAC2* was enhanced in ethylene-overproducing mutant *eto1-1*, but repressed in ethylene-insensitive mutants *etr1-1* and *ein2-1*, and auxin-insensitive mutant *tir1-1*. Overexpression of *AtNAC2* promoted lateral root development under both normal and salinity conditions (He et al., 2005). In *Arabidopsis*, NEK6, a NIMA-related kinases (NEKs), was induced by ACC and salinity. NEK6 positively regulated plant growth, seed yield and plant response to salinity and osmotic stresses, probably through suppression of ethylene biosynthesis and activation of cell division (Zhang et al., 2011a). In addition, many other stress-responsive factors, such as TINY (a GCC/DRE-binding protein-like transcription factor; Sun et al., 2008), AtMYB15 (a negative regulator of DREB1/CBF), RAB18 (an ABA responsive factor; Chen et al., 2014), and SIED1 (salt-induced and EIN3/EIL1-dependent 1; Peng et al., 2014a), were also found to be regulated by ethylene signaling and mediate ethylene-involved salinity responses.

Through these factors, ethylene-mediated salinity signaling was transferred into a series of nuclear transcriptional cascades which led to the expression changes of numerous stress-related effectors. Generally, these effectors could be classified into three major types according to their bio-functions in stress responses: (1) ROS scavengers such as SOD and POD (Kukreja et al., 2005; Peng et al., 2014a); (2) ion transporters such as HAK5 and HKTs (Moller et al., 2009; Jiang et al., 2013; Yang et al., 2015b); (3) osmolyte synthetic enzymes such as P5CS (Hsieh et al., 2013). Changes of these effectors usually led to physiological modification (such as homeostasis of ROS and Na^+^/K^+^) of plants for better adaptation to salinity condition.

## Interactions Between Ethylene and other Signals Under Salinity

In addition to directly regulating salinity-related effectors, ethylene also coordinates with some other phytohormones and stress signaling molecules to modulate plant response to salinity and normal growth. As an essential stress hormone, ABA participates in plant response to a series of biotic and abiotic stresses. Interactions between ABA and ethylene signaling in seed germination are extensively investigated. Ethylene ETR1-CTR1-EIN2 signaling suppressed ABA signaling in seeds, thus alleviated ABA-mediated inhibition of seed germination (Beaudoin et al., 2000). And NO was proposed to be an interactor between ethylene and ABA in seed (reviewed in Arc et al., 2013). The relationship between ethylene and ABA in salinity response is not as clear as in seed germination. Nevertheless, from ABA-associated expression pattern and mutant phenotypes of ethylene-related factors, we proposed that the whole ethylene biosynthesis and signal transduction pathway interacted with ABA in regulating salinity response. The expressions of *AtACS5*, *AtACS7*, *TaACO1*, *OsERF3*, *GmERF3*, *GhERF1*, and some other ethylene-related genes (Wang et al., 2005; Qiao et al., 2008; Zhang et al., 2009, 2013; Chen et al., 2014) were all regulated by ABA and salinity. Mutation of *ACS7* enhanced plant tolerance to salt, osmotic and heat stresses, possibly through elevating the expression of stress-responsive genes involved in ABA signaling under salinity (Dong et al., 2011). In *Arabidopsis*, *etr1* loss-of-function mutants showed reduced sensitivity to ABA and accelerated seed germination, while *etr2* loss-of-function mutants became more sensitive to ABA and germinated slower than the wild type, indicating contrasting roles of ETR1 and ETR2 in seed germination during salt stress. But this seemed to be mediated by affecting the ABA signaling but independent of ethylene signaling (Wilson et al., 2014). Disruption of EIN2 increased ABA level but substantially reduced the induction of *RD29B* under high salinity (Wang et al., 2007). Actually, the central ethylene signaling component EIN2 plays an important role in mediating the interactions between ethylene and several other hormones, including ABA.

In rice, MHZ4 (homologous to *Arabidopsis* ABA4) and MHZ5 (carotenoid isomerase), identified from *mhz* ethylene-response mutants, are involved in ABA biosynthesis (Ma et al., 2014; Yin et al., 2015). Mutation of either *MHZ4* or *MHZ5* reduced the ABA level but promoted ethylene production in etiolated seedlings. Ethylene treatment induced the expressions of *MHZ4* and *MHZ5*, thus increased the accumulation of ABA in roots. Loss-of-function mutants of *MHZ4* and *MHZ5* showed alleviated ethylene-inhibition of root growth, and this could be largely rescued by ABA treatment. Genetic analysis revealed that both MHZ4 and MHZ5-dependent ABA pathways acted downstream of ethylene receptors to positively regulate root response to ethylene (Ma et al., 2014; Yin et al., 2015). These findings first uncovered a different mechanism of controlling root growth in rice by ethylene-ABA interaction, while in *Arabidopsis* ABA is not necessary for ethylene to inhibit root growth. Given that ABA production and signaling are necessary for plant responses to salinity and other stresses, MHZ4 and MHZ5 are anticipated to have some roles in plant responses to salinity and other stresses. Thus, besides regulating seedling growth, MHZ4 and MHZ5 may also mediate the interaction between ethylene and ABA on controlling stress responses.

In addition, some other stress-related phytohormones are also related to ethylene in salinity response, including JA, SA (salicylic acid), BR (brassinosteroid), and so on. It was reported that the cross-effects between ethylene, JA, SA, and BR signaling pathways played important roles in plant defense response (Divi et al., 2010; Yang et al., 2013). They may function synergistically or antagonistically to precisely regulate defense responses. Loss-of-function of EIN2 eliminated plant response to JA, and expression of the EIN2-CEND was sufficient to recover this responsiveness, indicating that EIN2 is a molecular link between ethylene and JA signaling pathway (Alonso et al., 1999). It was shown that EIN3/EIL1-ERF1 might act as a node to integrate JA and ethylene signaling and regulate plant development as well as stress defense (Lorenzo et al., 2003; Zhu et al., 2011). In *Arabidopsis*, ERF1 played positive roles in plant tolerance to salinity, drought and heat stress by regulation of stress-related genes, which integrated the ethylene, JA and ABA signals (Cheng et al., 2013). In rice, the ethylene, JA and SA pathways are all involved in the induction of OsPR10 by stress, and OsERF1 may function in both ethylene and JA pathway to regulate this induction (Takeuchi et al., 2011). Ethylene insensitive mutant *ein2* exhibited hypersensitivity to salinity on seed germination. This hypersensitivity could be rescued by treatment with 24-epibrassinolide (EBR; Divi et al., 2010). Besides these stress-hormones, ethylene also interacts with auxin extensively on regulation of plant growth and development (reviewed in Vandenbussche et al., 2012). Recently, several auxin-related small proteins (SAURs and ARGOS) from *Arabidopsis* were found to act as brakes of ethylene signaling and as accelerators of cell proliferation and/or cell expansion to coordinate seedling growth and ethylene response (Li et al., 2015b; Rai et al., 2015). We propose that these proteins may also act as modulators in regulation of plant growth under salinity and other stresses.

Moreover, on regulation of salinity response, ethylene also interacts with many stress signaling molecules, including ROS and cGMP. In rice, salinity triggered MAPK cascades to stabilize ACSs, led to enhanced ethylene production and ethylene signaling, which then promoted ROS accumulation and growth inhibition (Li et al., 2014a; reviewed in Steffens, 2014). In *Arabidopsis*, salinity-induced EIN3/EIL1 conferred enhanced tolerance to salinity by promoting the ROS scavenging in an EIN2-independent manner (Peng et al., 2014a). Both ACC and cGMP treatments could promote the ethylene production and alleviate salinity-induced injury by homeostasis of Na^+^/K^+^. Further analysis based on the ethylene-insensitive mutant *etr1-3* and treatments with ethylene biosynthesis inhibitor and guanylate cyclase inhibitor revealed that cGMP modulated ethylene-mediated salinity response pathway by regulation of ethylene biosynthesis and perception (Li et al., 2014b).

## Novel Negative Feedback Mechanisms in Ethylene Signaling

**FIGURE 1:**
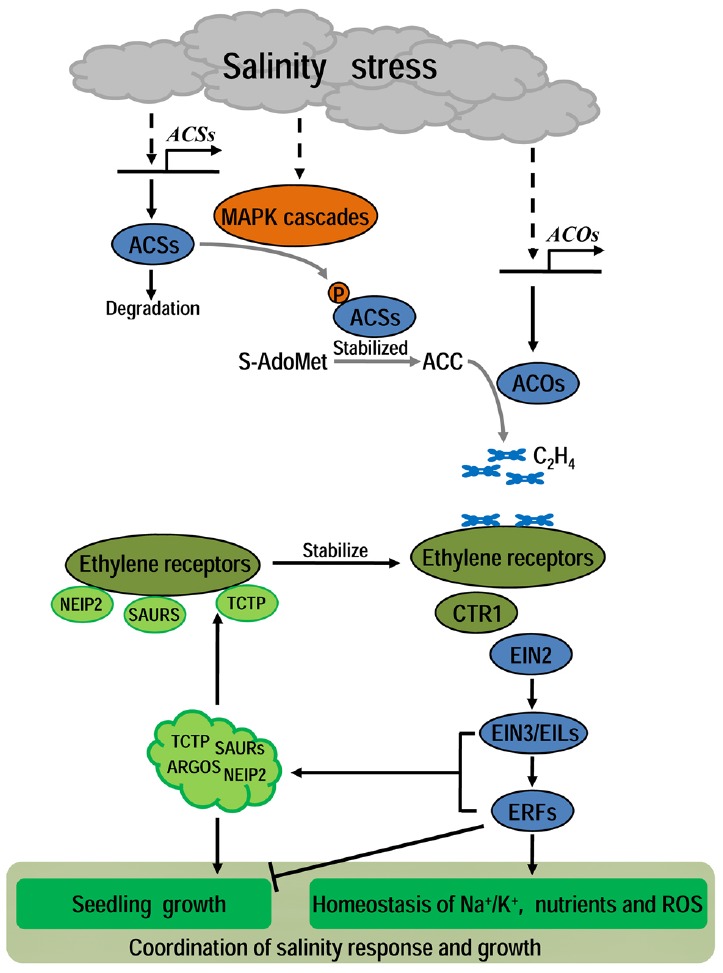
**A diagram of the ethylene’s role in plant under salinity.** Under salinity, stress signals could activate the MAPK phosphorylation cascades which then stabilize some ACSs to promote ethylene production. The transcripts level of *ACSs* and *ACOs* are also up-regulated under salinity. Then, salinity-induced ethylene signal is transduced mainly through the classical receptors-CTR1-EIN2-EIN3 pathway to regulate many effectors involved in plant growth and salinity response. Under proper concentration, ethylene promotes the homeostasis of Na^+^/K^+^, nutrients and ROS to enhance plant tolerance to salinity, with no irreversible inhibition of normal growth, while excessive ethylene will lead to harmful hyper-inhibition of plant growth. To avoid damage from extreme ethylene response, plants have evolved some negative feed-back mechanisms to alleviate ethylene response and promote seedling growth. In these mechanisms, some small proteins such as NtTCTP, NEIP2, AtSAURs, and AtARGOS are supposed to act as key modulators to coordinate plant growth and ethylene/salinity responses.

Generally speaking, ethylene is supposed to be a coordinator between stress response and growth. Intrinsic ethylene production and signaling is indispensable for plant rapid response to salinity and self-modification for better survival. But excessive ethylene production under continuous stress tends to largely inhibit plant growth and development, even leads to death. Therefore, tight control of ethylene homeostasis is critical for plants to survive under salinity and recover growth later. Indeed, there are multiple positive and negative feedback mechanisms for regulation of ethylene biosynthesis and signaling (reviewed in Vandenbussche et al., 2012). In addition, some ethylene and salinity-responsive small proteins were identified as restrictors in alleviating the ethylene-inhibited growth through suppression of ethylene response and promotion of cell proliferation and/or expansion. The most typical restrictors are NEIP2 and NtTCTP, two ethylene receptor NTHK1-interacting proteins in tobacco (Cao et al., 2015; Tao et al., 2015). Both NEIP2 and NtTCTP proteins were induced by ethylene treatment. Overexpressing either of them led to reduced sensitivity to ethylene and improved vegetative growth. Further investigation revealed that NtTCTP could stabilize the ethylene receptor NTHK1 and promote cell proliferation to reduce ethylene sensitivity and alleviate ethylene-inhibition of vegetative growth (Tao et al., 2015). NTHK1 phosphorylates NEIP2 *in vitro* and NEIP2 can be phosphorylated *in planta* in response to ethylene and salt treatment. Overexpression of NEIP2 improved plant tolerance to salt and oxidative stresses (Cao et al., 2015). Recently, two studies found that *ARGOS* (*auxin regulated gene involved in organ size*) genes in *Arabidopsis* were induced by ethylene dose-dependently, but this induction was suppressed in ethylene-insensitive mutants. Increasing the expression of ARGOS family members reduced seedling sensitivity to ethylene (Rai et al., 2015; Shi et al., 2015). More recently, we found that AtSAUR76/77/78 could associate with subfamily II ethylene receptor ETR2 and EIN4 to reduce ethylene response and promote plant growth (Li et al., 2015b). These findings reveal novel negative feedback mechanisms of precisely desensitizing excessive ethylene response but promoting growth recovery from ethylene-inhibited growth. All these small proteins are supposed to act as brakes of ethylene signaling and accelerators of cell proliferation/expansion to coordinate normal growth and ethylene response (Figure 1). Further investigation of the roles of these factors in ethylene-mediated response to salinity and other stresses would be valuable for elucidating the overall roles of ethylene in modulation of plant growth under environmental stresses.

Considering the key role of NEIP2-type factors in constraint of ethylene signaling, gene manipulation of these factors would be an effective way to enhance plant tolerance to salinity stress. This might be achieved by using salinity-responsive promoters. A previous study showed that distinctive expression of HKT1;1 in the root stele of *Arabidopsis* reduced root-to-shoot Na^+^ delivery, thus promoting seedling tolerance to salinity, while constitutive expressing HKT1;1 increased Na^+^ accumulation in shoot and led to more sensitive to salinity (Moller et al., 2009). In addition, it was shown that ethylene promoted salinity tolerance largely through improving the homeostasis of Na^+^/K^+^(Jiang et al., 2013). These findings suggest another way to enhance salinity tolerance of plants by cell type-specific engineering of ethylene signaling.

## Cross-Talks Between Responses to Salinity and Responses to Nutrient Deficiencies

Besides ion toxicity, osmotic and oxidative stresses, high salinity also led to nutrient deficiencies (Chinnusamy et al., 2006). The most direct salinity-related nutrient is potassium (K), which has critical roles in maintaining enzyme activities, ion homeostasis, internal pH, and etc. For similar chemical feature, excessive sodium ions inhibit potassium uptake and lead to potassium deficiency. Usually, high concentration of sodium and low concentration of potassium status (high sodium/potassium ratio) is most harmful for plant cells. Similar to the vital roles of ethylene in high salinity response, ethylene also played important roles in response to potassium deficiency and acted upstream of ROS (Jung et al., 2009). One piece of evidence was that the low K^+^-inducible *HAK5* expression was dependent on ethylene signaling (Jung et al., 2009). In addition, changes of ethylene biosynthesis and signaling often lead to alteration of sodium/potassium contents under salinity. In tobacco, ethylene receptor *NTHK1*-overexpressing tobacco seedlings showed higher sodium/potassium ratio than the WT (Cao et al., 2006). In fact, Na^+^/K^+^ homeostasis is the key point for ethylene regulation of plant salinity response (Jiang et al., 2013).

In addition to potassium deficiency, ethylene also regulates plant responses to many other nutrient (N, P, Ca, and Fe) deficiencies through different pathways (reviewed in García et al., 2015). Among them, the most frequently studied is iron (Fe) deficiency. Previous works showed that ethylene participated in up-regulation of many important Fe-regulated genes in Strategy I plants, including ferric reductase, H^+^-ATPase gene and several Fe acquisition genes *FIT*, *FRO2*, and *IRT1* (Lucena et al., 2006; Waters et al., 2007; García et al., 2010). Simultaneously, Fe deficiency promoted the expressions of many genes related to ethylene biosynthesis and signaling in the roots (García et al., 2010). Moreover, recent work showed that hypoxia and bicarbonate, two main factors causing Fe chlorosis in Strategy I plants, negatively regulated the expressions of Fe acquisition genes, probably by affecting ethylene synthesis and/or signaling (García et al., 2014). Meanwhile, salinity stress usually reduced the Fe uptake and led to Fe deficiency (Rabhi et al., 2007; Yousfi et al., 2007). Considering the positive roles of ethylene in both salinity tolerance and Fe acquisition, we can deduce that ethylene mediates the cross-talk between salinity and Fe deficiency responses, possibly through regulation of many genes involved in Fe homeostasis under salinity condition.

Another focused research field is the cross-talk between phosphate (P) deficiency and ethylene, and a simple model has been supposed (Lei et al., 2011b). First, P starvation might induce ethylene production and enhance plant sensitivity to ethylene (He et al., 1992; Borch et al., 1999). Then enhanced ethylene production and ethylene responses would promote the development of root hair and the expression of *PSI* (*P starvation-induced*) genes (Lei et al., 2011b). These changes could directly affect P uptake, remobilization and redistribution, which facilitate plants to maintain P homeostasis under P-deficient condition. Interestingly, ethylene alone seemed to be not enough to promote the *PSI* gene expression to the degree induced by P starvation, suggesting a cross-talk between ethylene and P-deficiency-induced signals in controlling the *PSI* gene expression under low P (Lei et al., 2011b). Moreover, it was found that inhibition of ethylene biosynthesis or signaling could respectively rescued the increased primary root elongation and root hair formation caused by overexpression of the P transporter gene *Pht1;5*, providing another evidence of cross-talk between ethylene and P signaling (Nagarajan et al., 2011). Indeed, ethylene plays an integrative role in regulating both local P-deficiency responses and systemic P signaling pathways (reviewed in Nagarajan and Smith, 2012). Usually, high salinity led to reduced P uptake (Rai and Sharma, 2006), and phosphate-accumulating mutants *siz1* and *pho2* showed reduced uptake and reduced accumulation of Na^+^, hence enhanced plant tolerance to salinity stress (Miura et al., 2011). All these studies indicate that ethylene may mediate the cross-talk between salinity and P deficiency stresses.

Recently, the cross-talk between nitrate (N) deficiency and ethylene was investigated. Low nitrate treatment rapidly induced ethylene production and up-regulated the expression of *EIN3/EIL1* and *NRT2.1*, while enhanced ethylene production and signaling down-regulated the expression of *NRT2.1* and thus decreased the high-affinity nitrate uptake, indicating a negative feedback regulation of nitrate acquisition by ethylene under nitrate deficiency (Zheng et al., 2013). More recently, it was reported that cadmium and sodium stresses-induced ethylene and JA signaling converged at EIN3/EIL1 to up-regulate *NRT1.8* expression but down-regulate *NRT1.5* expression, thus mediated the stress-initiated nitrate allocation to roots (SINAR), which decoupled nitrate assimilation and photosynthesis, and finally decreased plant growth but promoted plant tolerance to stress in a nitrate reductase-dependent manner (Zhang et al., 2014).

All these findings suggest that ethylene mediates the cross-talks between plant response to salinity and responses to nutrient deficiencies. Ethylene may be a linking point in regulation of nutrient homeostasis under salinity stress to coordinate stress response and normal growth.

## Conclusion and Perspectives

From above studies, a general conclusion could be made that inherent ethylene production is necessary for the establishment of salt acclimation, and ethylene signaling is indispensable for plant self-adjustment in rapid response to salinity stress and better adaptation to the stress condition (summarized in Figure 1).

**FIGURE 2:**
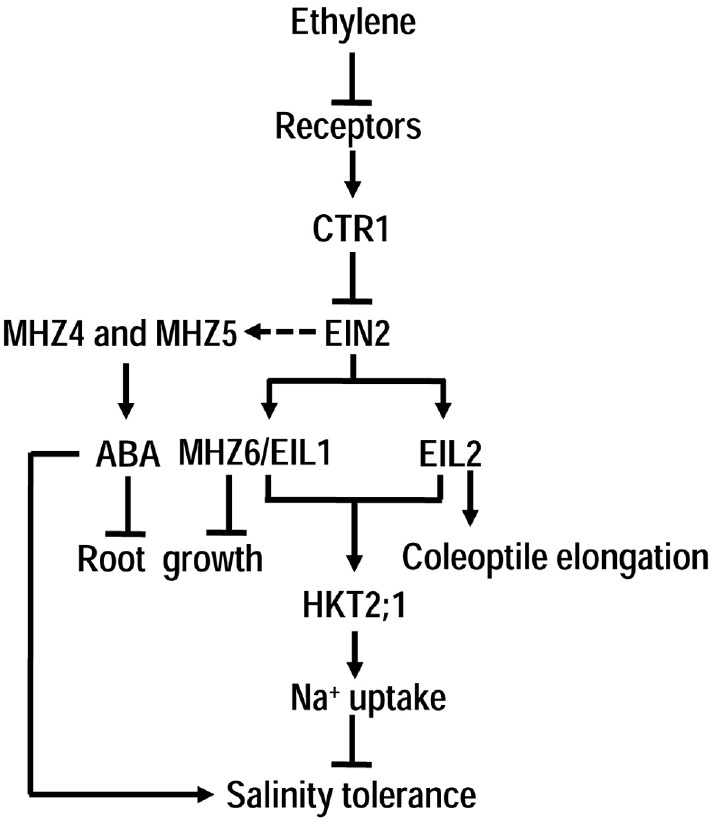
**The role of ethylene signaling in rice under salinity.** Unlike in *Arabidopsis*, the classical CTR1-EIN2-EILs ethylene signaling in rice plays negative role in regulating plant tolerance to salinity mainly through up-regulation of OsHKT2;1, a high affinity Na^+^ transporter with no homolog in *Arabidopsis*. Interestingly, EIL1 and EIL2 in rice respectively regulates ethylene-controlled roots and coleoptiles growth of etiolated seedlings. Besides, two novel ethylene signaling components MHZ4 and MHZ5 in rice may mediate the interaction between ethylene and ABA in regulation of root growth and salinity response.

Different from *Arabidopsis*, ethylene signaling in rice seems to be more complex, so does the ethylene’s role in salinity response (Figure 2). Ethylene treatment of rice seedlings increased salinity sensitivity, while 1-MCP (a blocker of ethylene perception) treatment led to enhanced tolerance to salinity. Rice MHZ7/OsEIN2, MHZ6/OsEIL1 and OsEIL2 conferred reduced tolerance to salinity (Yang et al., 2015b). MHZ6/OsEIL1 and OsEIL2 could bind to the promoter region of *OsHKT2;1* (encoding a Na^+^ transporter) and activated its expression for Na^+^ uptake. Ethylene-induced *OsHKT2;1* expression and Na^+^ uptake may represent a mechanism for maintaining ion homeostasis in water environment (Yang et al., 2015b). This mechanism could lead to sensitive response especially under high salinity. Different mechanisms in *Arabidopsis* and rice in response to salt stress may arise from the evolutionary divergence under different growing conditions or due to different plant species.

Although much progress has been made in terms of ethylene roles in salt stress responses, there are still some uncertain points left to be clarified. First, whether the functions of ACSs and ACOs on salinity response must be executed through downstream ethylene signaling? This could be solved directly through construction and analysis of double loss-of-function mutants between ACS and EIN2 or EIN3. Second, a few studies showed that enhanced ethylene production led to salinity sensitivity (Xu et al., 2008; Li et al., 2014a), but whether this sensitivity arises from inherent weakened salinity resistance or just from ethylene-inhibited growth and promoted senescence is unclear. This could be clarified through alleviating the effects of ethylene on plant growth and development by genetic method. Third, the appropriate ethylene quantity and signaling intensity for plant response to salinity may be varied during different growing stages. Thus, precise control of ethylene production and signaling may be critical for promotion of plant salinity tolerance. This could be achieved by gene manipulation of key ethylene biosynthesis and signaling factors using specific promoters. Last, further investigation of the differences between rice and *Arabidopsis* on the role of ethylene in salinity response is valuable for elucidating the evolutionary divergence of ethylene in different plant species. Additionally, identification of more ethylene-response mutants and evaluation of their salt response may reveal novel components linking ethylene and salt stress. Analysis of sequence variations of known components among different cultivars may identify better alleles for salt tolerance. Uncovering these problems will largely broaden our horizon and enrich our knowledge on how plant self-adjust to coordinate external stresses and internal growing motivation for better survival. This will be helpful for precisely controlling ethylene production and signaling to enhance salinity tolerance and improve agronomic traits of crops.

## Author Contributions

JJT, HWC, SYC, and JSZ conceived the topic. JJT wrote the manuscript. All authors revised the manuscript.

### Conflict of Interest Statement

The authors declare that the research was conducted in the absence of any commercial or financial relationships that could be construed as a potential conflict of interest.
